# Herpes simplex virus and rates of cognitive decline or whole brain atrophy in the Dominantly Inherited Alzheimer Network

**DOI:** 10.1002/acn3.51669

**Published:** 2022-10-03

**Authors:** Charlotte Warren‐Gash, Sharon L. Cadogan, Jennifer M. Nicholas, Judith M. Breuer, Divya Shah, Neil Pearce, Suhail Shiekh, Liam Smeeth, Martin R. Farlow, Hiroshi Mori, Brian A. Gordon, Georg Nuebling, Eric McDade, Randall J. Bateman, Peter R. Schofield, Jae‐Hong Lee, John C. Morris, David M. Cash, Nick C. Fox, Basil H. Ridha, Martin N. Rossor, for the Dominantly Inherited Alzheimer Network

**Affiliations:** ^1^ Department of Non‐Communicable Disease Epidemiology London School of Hygiene and Tropical Medicine London United Kingdom; ^2^ Department of Medical Statistics London School of Hygiene and Tropical Medicine London United Kingdom; ^3^ Institute of Child Health University College London Gower Street London WC1E 6BT United Kingdom; ^4^ Virology Department Great Ormond Street Hospital London United Kingdom; ^5^ Indiana University School of Medicine Indianapolis Indiana USA; ^6^ Department of Clinical Neuroscience Osaka Metropolitan University Medical School, Sutoku University Osaka Japan; ^7^ Department of Radiology Washington University School of Medicine in St Louis Missouri USA; ^8^ German Center for Neurodegenerative Diseases Site Munich Germany; ^9^ Department of Neurology Ludwig‐Maximilians University Munich Germany; ^10^ Department of Neurology Washington University School of Medicine St. Louis USA; ^11^ Neuroscience Research Australia Sydney New South Wales Australia; ^12^ School of Medical Sciences University of New South Wales Sydney New South Wales Australia; ^13^ Department of Neurology University of Ulsan College of Medicine, Asan Medical Center Seoul South Korea; ^14^ UK Dementia Research Institute University College London London United Kingdom; ^15^ Dementia Research Centre, Institute of Neurology University College London Queen Square London United Kingdom; ^16^ NIHR University College London Hospitals Biomedical Research Centre London United Kingdom

## Abstract

**Objective:**

To investigate whether herpes simplex virus type 1 (HSV‐1) infection was associated with rates of cognitive decline or whole brain atrophy among individuals from the Dominantly Inherited Alzheimer Network (DIAN).

**Methods:**

Among two subsets of the DIAN cohort (age range 19.6–66.6 years; median follow‐up 3.0 years) we examined (i) rate of cognitive decline (*N* = 164) using change in mini‐mental state examination (MMSE) score, (ii) rate of whole brain atrophy (*N* = 149), derived from serial MR imaging, calculated using the boundary shift integral (BSI) method. HSV‐1 antibodies were assayed in baseline sera collected from 2009–2015. Linear mixed‐effects models were used to compare outcomes by HSV‐1 seropositivity and high HSV‐1 IgG titres/IgM status.

**Results:**

There was no association between baseline HSV‐1 seropositivity and rates of cognitive decline or whole brain atrophy. Having high HSV‐1 IgG titres/IgM was associated with a slightly greater decline in MMSE points per year (difference in slope − 0.365, 95% CI: −0.958 to −0.072), but not with rate of whole brain atrophy. Symptomatic mutation carriers declined fastest on both MMSE and BSI measures, however, this was not influenced by HSV‐1. Among asymptomatic mutation carriers, rates of decline on MMSE and BSI were slightly greater among those who were HSV‐1 seronegative. Among mutation‐negative individuals, no differences were seen by HSV‐1. Stratifying by *APOE4* status yielded inconsistent results.

**Interpretation:**

We found no evidence for a major role of HSV‐1, measured by serum antibodies, in cognitive decline or whole brain atrophy among individuals at high risk of early‐onset AD.

## Introduction

Whether chronic neurotropic viruses such as herpes simplex virus type 1 (HSV‐1) play a causal role in Alzheimer's disease (AD) and other dementias remains controversial. Widespread deposition of amyloid‐β, a pathological hallmark of AD, occurs in response to infection of human neuronal cell cultures and AD animal models with herpesviridae^1^, ^2^ and may form part of the innate immune response to infections in the central nervous system.^3^ Hyperphosphorylation of tau protein, which forms another key pathological feature of AD, may also be induced by HSV‐1^4^, ^5^ and by the less common infection HSV‐2.^6^ Nevertheless, longitudinal studies investigating the relationship between HSV‐1 antibodies and risk of clinical outcomes such as cognitive decline or incident AD have shown mixed results.^7^, ^8^, ^9^, ^10^, ^11^, ^12^

Two community studies of ageing cohorts from the United States showed no association between HSV‐1 seropositivity (representing ever infection) or antibody titres (which may reflect recent viral infection or reactivation) and cognitive decline, although they noted that antibodies against cytomegalovirus (CMV), another herpesvirus, were associated with a more rapid decline in global cognition over time.^7^, ^8^ This finding was recently confirmed using data from the US Health and Retirement Study.^10^ A cohort study from Rotterdam reported subtle cognitive disturbance including global cognition and cognitive domains of memory, information processing and executive function among individuals with higher HSV‐1 antibody titres, although HSV‐1 was not associated with AD risk over 9 years of follow‐up.^9^ Two other cohorts which included HSV‐1 seropositivity as part of an infectious burden index along with HSV‐2 and CMV^11^ and HSV‐2, CMV, *Helicobacter pylori* and *Chlamydia pneumoniae* ^12^ showed that a higher infectious burden was associated with cognitive decline among older people.

Other studies have focussed on interactions between HSV‐1 and genetic risk factors for AD, notably the *APOE4* allele: murine studies suggest that *APOE4* facilitates HSV‐1 latency in the brain compared to *APOE3* or *APOE* absence in knockout mice.^13^ In a French cohort, having high HSV‐1 IgG titres or IgM was associated with increased AD risk only among individuals who were *APOE4* positive.^14^ Further data from this and another cohort showed that HSV‐1 seropositivity was correlated with more white matter alterations in the parahippocampal cingulum and fornix as well as lower hippocampal volumes for those with the highest HSV‐1 tertiles only, while AD risk was only associated with HSV‐1 for *APOE4* carriers.^15^ It is not known whether HSV‐1 interacts with other genetic risk factors such as autosomal dominant mutations in presenilin‐1 (*PSEN1),* presenilin‐2 *(PSEN2)* and amyloid precursor protein *(APP)*, which cause early‐onset AD, to influence disease progression.

We therefore aimed to investigate whether HSV‐1 seropositivity, indicating ever exposure to HSV‐1, or high IgG antibody titres and/or IgM antibodies, indicating recent HSV‐1 infection or reactivation, were associated with rates of cognitive decline or whole brain atrophy among individuals with and without *PSEN1*, *PSEN2* and *APP* mutations from the Dominantly Inherited Alzheimer Network (DIAN) cohort.

## Methods

### Approvals

This study was approved by the DIAN Steering Committee (ref DIAN‐T1504, date: 06/07/2016). Ethical approval was obtained from the National Health Service Health Research Authority South Central – Berkshire Research Ethics Committee through a substantial amendment to the original DIAN application (original ref 09/H0505/73; amendment 11 (AM15), dated 03 July 2017), and from the London School of Hygiene & Tropical Medicine research ethics committee (ref 14601).

### Data source and study population

DIAN is an international prospective cohort study established in 2008, which is described in detail elsewhere.^16^, ^17^ In brief, DIAN participants are adult biological children of an affected parent with an autosomal dominant *APP, PSEN1* or *PSEN2* mutation causing early‐onset autosomal dominant AD (ADAD). DIAN participants include both symptomatic and asymptomatic mutation carriers as well as non‐carrier family members. All cohort participants were invited to complete clinical and cognitive assessments including mini‐mental state exam (MMSE) at baseline and at clinic visits initially held yearly for symptomatic participants and every 2 or 3 years for asymptomatic participants. At clinic visits, blood and cerebrospinal fluid (CSF) samples were also taken and tested for a range of biomarkers including genetics, while neuroimaging was conducted using methods including MRI, Fluorodeoxyglucose positron emission tomography (PET) and Pittsburgh Compound B amyloid PET. This study used data and biospecimens from DIAN Datafreeze 11.

### Exposure definition

We considered the following binary exposures: (1) HSV‐1 seropositivity defined using HSV‐1 IgG status at baseline (to represent ever infection); (2) HSV‐1 high titres/IgM defined as either HSV‐1 IgG titre in the top tertile among those testing positive or the presence of IgM antibodies measured at baseline (to represent recent infection or reactivation).

We used stored serum samples from DIAN participants taken at baseline and each follow‐up visit and frozen at −80°C. Aliquots of samples were extracted from storage for testing in January 2018. A quantitative commercially available HSV‐1 immunoassay, the RIDASCREEN® HSV 1 + 2 IgG kit and IgM, was used to test serum samples for HSV‐1 antibodies. This enzyme immunoassay works on the principle of binding of HSV‐type‐specific antibodies present in the serum to antigen coated on the microwell plate. This is measured by the addition of enzyme‐labelled anti‐human antibodies (conjugate), which convert colourless substrate to a blue end product. This enzymatic reaction is measured as optical density by stopping the reaction that changes the colour of the end product from blue to yellow. The results are determined by measuring the optical density (OD) with an automated ELISA plate reader. Each test run was verified for validity by following the criteria provided in each kit and evaluated using the standard curve provided. The range for limit of quantification by RIDASCREEN® was 10–1000 U/mL. We used standard cut‐offs to quantify HSV‐1 IgG serostatus as positive, negative or equivocal, but dropped those with an equivocal status for analysis (*N* = 6).

### Outcome definition

The outcomes were (i) rate of cognitive decline measured by change in the MMSE score and (ii) whole brain atrophy (measured in mL) derived from serial MR imaging. Whole brain volume was measured at each MRI scan and the boundary shift integral (BSI) was calculated to assess atrophy between scans using the gBSI method.^18^ For inclusion, participants needed to have at least two measurements of either outcome to calculate a rate of change, taken after a valid HSV‐1 measurement. Participants with multiple measures had outcomes updated at each new measurement.

### Covariates

Covariates were measured at cohort entry and included sex (male, female), age, education level (below high school, graduated high school, some college education, bachelor's degree or above), weight status (normal or underweight, overweight, defined using classic cut offs for body mass index), race (white, non‐white), smoking status (never/ever), alcohol abuse, diabetes, hypertension, *APOE4* (absent/present = either heterozygous or homozygous), mutation status (positive/negative) and symptom status (symptomatic defined as Clinical Dementia Rating (CDR) >0 /asymptomatic defined as CDR = 0). A combined mutation and symptom status variable was derived from the mutation and symptom status variables and categorised as (i) mutation negative (ii) mutation positive, asymptomatic and (iii) mutation positive, symptomatic.

### Statistical analysis

We first described baseline characteristics of the overall cohort by HSV‐1 status, and compared these with characteristics of those eligible for inclusion in the samples for longitudinal analyses of MMSE and BSI outcomes. For the longitudinal analysis, we used mixed‐effects linear regression models to compare rates of (a) cognitive decline and (b) whole brain atrophy between individuals with and without evidence of HSV‐1 seropositivity. These initial models were not stratified by mutation or symptom status to maximise study power. The mixed model for cognitive decline included terms for the exposure, covariates and continuous time (years since the baseline visit) to account for associations with initial MMSE scores, and also interaction terms for the exposure and covariates with continuous time to estimate the rate of change (slope) in cognition over the time period. The model for atrophy included continuous time and interaction terms for exposure and covariates with continuous time (years since the baseline MRI scan), to estimate the effects of each variable on atrophy rate. We adjusted for the following potential confounders in the models for cognitive decline: age, sex, educational level, race and smoking status. Smoking status was dropped from the BSI analysis to maximise power. Random effects were included for the intercept and slope (time in study, in years). We also considered mutation and symptom status and *APOE4* status as effect modifiers by fitting separate models for each variable that included an interaction between the variable and HSV‐1 seropositivity and a three‐way interaction between the variable and HSV‐1 seropositivity and time. Using these models, we presented fully adjusted effects of HSV‐1 exposure stratified by each effect modifier separately. We then repeated the above analyses for the exposure HSV‐1 high titres/IgM. For the cognitive decline outcome, MMSE scores were negatively skewed; therefore, bootstrapping (2000 replications) was used to provide bias‐corrected and accelerated bootstrap confidence intervals (CIs). All analyses were carried out in Stata version 16 MP (Statacorp).

## Results

### Overview of participants

Baseline characteristics of the overall cohort (*N* = 387) and samples included in longitudinal analyses for MMSE (*N* = 164) and BSI outcomes (*N* = 149) are shown in Table 1 by HSV‐1 serology status at the first measurement. Overall, 220 participants (56.9%) were HSV‐1 seropositive and 138 (35.7%) had evidence of either high IgG titres or IgM. Those who were seropositive or had high titres/IgM were slightly older, had lower levels of education and were more likely to be symptomatic at cohort entry. The median age was 40.7 years (IQR: 33.9–49.0) for seropositives compared to 36.8 years (IQR 29.0–45.7) among those who were seronegative, and 41.3 years (IQR 33.6–49.0) for those with high titre IgG/IgM compared to 38.1 years (IQR 30/2–46.9) among those without high titre IgG/IgM.

**Table 1:** Baseline characteristics of the overall cohort and study samples included in longitudinal analyses for MMSE and BSI outcomes, by HSV‐1 infection status *N* (%).

	Overall cohort (*N* = 387)		MMSE sample (*N* = 164)		BSI sample (*N* = 149)	
	HSV‐1 seropositive	HSV‐1 high titres/IgM	HSV‐1 seropositive	HSV‐1 high titres/IgM	HSV‐1 seropositive	HSV‐1 high titres/IgM
	*N* = 220 (56.8)	*N* = 138 (35.7)	*N* = 105 (64.0)	*N* = 62 (37.8)	*N* = 91 (61.1)	*N* = 54 (36.2)
Sex						
Female	126 (57.5)	83 (38.0)	61 (60.4)	37 (36.6)	51 (56.0)	36 (40.0)
Male	94 (56.0)	55 (32.7)	44 (69.8)	25 (39.6)	40 (69.0)	18 (31.0)
Age category						
<30 years	29 (37.2)	20 (25.6)	10 (50.0)	4 (20.0)	9 (40.9)	8 (36.4)
30–35 years	34 (57.6)	19 (32.2)	14 (73.7)	9 (50.0)	15 (68.2)	8 (36.4)
35–40 years	39 (58.2)	23 (34.3)	18 (66.7)	8 (29.6)	14 (58.3)	8 (33.3)
40–45 years	37 (63.8)	27 (46.6)	19 (61.3)	15 (46.9)	14 (60.9)	8 (34.8)
45–50 years	35 (64.8)	21 (38.9)	19 (65.5)	13 (44.8)	17 (63.0)	10 (37.0)
>50 years	46 (64.8)	28 (39.4)	25 (65.8)	13 (34.2)	22 (71.0)	12 (38.7)
Race						
White	164(56.9)	99 (34.4)	79 (62.7)	47 (37.3)	70 (57.9)	42 (34.7)
Non‐White	22 (52.4)	17 (40.5)	13 (61.9)	10 (47.6)	10 (62.5)	9 (56.2)
Missing	34 (56.9)	22 (38.6)	13 (76.5)	5 (29.4)	11 (91.7)	3 (25.0)
Education						
Below high school	36 (78.3)	17 (37.0)	15 (79.0)	9 (47.4)	16 (94.1)	8 (47.1)
Graduated high school	50 (65.8)	31 (40.8)	27 (77.1)	16 (45.7)	22 (71.0)	13 (41.9)
Some college education	64 (57.1)	37 (33.0)	27 (58.7)	11 (23.9)	23 (51.1)	11 (24.4)
Bachelor's degree or above	70 (45.8)	53 (34.6)	36 (56.3)	26 (40.6)	30 (53.4)	22 (39.3)
Weight status						
Normal or underweight	56 (54.9)	30 (29.4)	34 (60.7)	16 (34.8)	28 (62.2)	14 (31.1)
Overweight	104 (53.4)	67 (37.6)	58 (63.0)	29 (40.9)	40 (55.6)	27 (37.5)
Missing	60 (56.1)	41 (38.2)	13 (81.3)	17 (36.2)	23 (71.9)	13 (40.6)
Smoking status						
Never smoked	109 (54.2)	69 (34.3)	60 (64.5)	34 (37.8)	54 (62.8)	33 (38.4)
Ever smoked	90 (59.6)	56 (37.1)	45 (63.4)	26 (37.7)	35 (57.4)	20 (33.9)
Missing	21 (60.0)	13 (37.1)	0 (0.0)	2 (40)	2 (100)	1 (25.0)
Alcohol abuse	14 (58.3)	7 (29.2)	5 (71.4)	2 (50.0)	5 (71.4)	1 (14.3)
Hypertension	26 (60.5)	20 (46.5)	14 (73.7)	8 (44.4)	7 (58.3)	5 (41.7)
Diabetes	7 (58.3)	6 (50.0)	2 (100)	0 (0.0)	1 (1.1)	1 (33.3)
Symptom status						
Asymptomatic	148 (52.5)	93 (33.0)	62 (59.1)	36 (34.3)	54 (54.6)	34 (34.3)
Symptomatic	72 (68.6)	45 (42.9)	43 (72.9)	26 (44.1)	37 (74.0)	20 (40.0)
*APOE4* allele present	66 (59.5)	45 (40.6)	33 (61.1)	21 (38.9)	27 (60.0)	17 (37.8)
Mutation positive	137 (58.6)	86 (36.8)	72 (65.5)	44 (40.0)	65 (65.0)	37 (37.0)

### Association between HSV‐1 serology and rate of change in MMSE scores

There was no association between HSV‐1 seropositivity and mean rate of change in MMSE scores (Table 2). There was a slightly greater rate of decline in the MMSE among those with high HSV‐1 IgG titres or IgM present compared to those without (difference in slope − 0.365 points per year, 95% CI: −0.958 to −0.072). Full model results are shown in Table S1. Table 3 shows the association between HSV‐1 serology status and rate of change in MMSE scores, stratified by mutation and symptom status and *APOE4* allele status.

**Table 2:** Association between HSV‐1 seropositivity and high antibody titres/IgM and the rate of change in MMSE scores.

Exposure	Mean rate of change^1^ (95% CI) unexposed	Mean rate of change^1^ (95% CI) exposed	Crude effect estimate^2^ (95% CI) *n* = 164	Fully adjusted effect estimate^2^ (95%CI) *n* = 147
HSV‐1 seropositivity	−0.678 (−1.064 to −0.369)	−0.718 (−1.029 to −0.456)	0.040 (−0.459 to 0.424)	0.021 (−0.438 to 0.520)
High antibody titres/IgM	0.582 (−0.799 to −0.265)	−0.890 (−1.430 to −0.566)	0.308 (−0.909 to 0.024)	−0.365 (−0.958 to −0.072)

^1^ Mean rate of change in MMSE, points per year.

^2^ Effect estimate is the difference in mean rates of change in MMSE (points per year) for the exposure versus unexposed group.

**Table 3:** Association between HSV‐1 seropositivity/high HSV‐1 antibody titres and rate of change in MMSE scores—by mutation and symptom status and *APOE4* status at baseline(*N* = 147).

Strata	Mean rate of change^1^ (95% CI) unexposed within strata	Mean rate of change^1^ (95% CI) exposed within strata	Fully adjusted effect estimate^2^ within strata (95%CI)
HSV‐1 seropositivity			
Mutation negative	0.036 (−0.123 to 0.224)	0.221 (0.008 to 0.466)	0.185 (−0.116 to 0.481)
Mutation positive, asymptomatic	−0.355 (−0.982 to 0.041)	0.135 (−0.047 to 0.320)	0.490 (0.081 to 1.135)
Mutation positive, symptomatic	−2.484 (−3.854, −0.950)	−2.333 (−3.240, −1.684)	0.151 (−1.812, 1.528)
*APOE4* Allele absent	−0.437 (−0.851, −0.034)	−0.578 (−1.001, −0.208)	−0.141(−0.732, 0.362)
*APOE4* Allele present	−0.737 (−1.496, −0.161)	−0.402 (−0.849, 0.164)	0.335 (−0.357, 1.350)
High HSV‐1 antibody titres/IgM			
Mutation negative	0.080 (−0.034 to 0.298)	0.027 (−0.161 to 0.478)	0.194 (−0.262 to 0.399)
Mutation positive, asymptomatic	0.043 (−0.191 to 0.159)	−0.280 (−0.865 to 0.313)	−0.323 (−0.922 to 0.399)
Mutation positive, symptomatic	−2.318 (−3.104 to −1.260)	−2.401 (−3.860 to −1.571)	−0.084 (−2.027 to 0.399)
*APOE4* Allele absent	−0.262 (−0.459 to 0.0261)	−0.922 (−1.593 to −0.455)	−0.659 (−1.480 to −0.242)
*APOE4* Allele present	−0.589 (−1.00 to 0.076)	−0.356 (−1.242 to 0.195)	0.233 (−1.059 to 0.828)

Bias‐corrected and adjusted 95% CIs are presented.

^1^ Mean rate of change in MMSE, points per year.

^2^ Effect estimate is the difference in mean rates of change in MMSE (points per year) for the exposure versus unexposed group.

#### HSV‐1 seropositivity and rate of change in MMSE scores

Among mutation‐positive, symptomatic individuals, MMSE scores declined fastest overall. However, there was no difference in the mean rate of MMSE change between those who were HSV‐1 seropositive and seronegative: 0.151 points per year (−1.812 to 1.528) in this group. Similarly, among mutation‐negative individuals, mean rate of MMSE change was not affected by HSV‐1 status, difference between seropositive and seronegative individuals: 0.185 points per year (−0.116 to 0.481).

Among mutation‐positive, asymptomatic individuals, the decline in MMSE score was greater for those who were HSV‐1 seronegative (−0.355 points per year (−0.982 to 0.041)) than those who were HSV‐1 seropositive (0.135 points per year (−0.047 to 0.320)). Overall, among mutation positive, asymptomatic individuals, the difference in the mean rate of MMSE change between those who were seropositive and seronegative was 0.490 points per year (0.081 to 1.135).

Among individuals with the *APOE4* allele (regardless of mutation/symptom status), there was no significant difference in the mean rate of MMSE change between those who were HSV‐1 seropositive and seronegative: 0.335 points per year (−0.357, 1.350). Similar results were seen among those without the *APOE4* allele: −0.141 points per year (−0.732, 0.362).

#### High HSV‐1 antibody titres/IgM and rate of change in MMSE scores

No differences were found in the mean rate of MMSE change between those who had high HSV‐1 antibodies/IgM and those who did not among mutation‐negative individuals (0.194 points per year (−0.262 to 0.399)), mutation‐positive, asymptomatic individuals (−0.323 points per year (−0.922 to 0.399)) or mutation‐positive, symptomatic individuals (−0.084 points per year (−2.027 to 0.399)).

Some differences were observed by *APOE4* allele status: among individuals without *APOE4*, the mean rate of change in MMSE score was −0.262 points per year (−0.459 to 0.0261) for those who had high HSV‐1 antibody titres/IgM, compared to −0.922 points per year for those who did not, which represented a significant difference of −0.659 points per year (−1.480 to −0.242). However, among individuals with the *APOE4* allele, the mean rate of MMSE change did not differ by HSV‐1 antibodies/IgM exposure status: 0.233 points per year (−1.059 to 0.828).

### Association between HSV‐1 infection on rate of whole brain atrophy, defined using mean BSI

There was no association between either HSV‐1 seropositivity or high antibody titres/IgM and rate of whole brain atrophy overall (Table 4; Table S2). Table 5 shows the association between HSV‐1 seropositivity/high HSV1 antibody titres and the rate of whole brain atrophy, by mutation and symptom status, and *APOE4* status.

**Table 4:** Association between HSV‐1 seropositivity and high antibody titres/IgM and the rate of whole brain atrophy (defined using mean BSI).

Exposure	Mean rate of change^1^ (95% CI) unexposed	Mean rate of change^1^ (95% CI) exposed	Crude effect estimate^2^ (95% CI) *n* = 149	*p*‐value	Fully adjusted effect estimate^2^ (95%CI) *n* = 137	*p*‐value
HSV‐1 seropositivity	3.334 (1.844 to 4.823)	3.904 (2.614 to 5.193)	0.570 (−1.400 to 2.540)	0.571	−1.174 (−3.230 to 0.892)	0.265
High antibody titres/IgM	3.641 (2.423 to 4.859)	3.696 (2.058 to 5.334)	0.055 (−1.986 to 2.096)	0.958	0.703 (−1.339 to 2.745)	0.500

^1^ Mean rate of change in BSI, mL per year.

^2^ Effect estimate is the difference in mean rates of change in BSI (mL per year) for the exposure versus unexposed group.

**Table 5:** Association between HSV‐1 seropositivity and rate of whole brain atrophy (defined using BSI)—by mutation and symptom status and *APOE4* status at baseline (*N* = 137).

Strata	Mean rate of change^1^ (95% CI) unexposed within strata	*p*‐value	Mean rate of change^1^ (95% CI) exposed within strata	*p*‐value	Fully adjusted effect estimate^2^ within strata (95%CI)	*p*‐value
HSV‐1 seropositivity						
Mutation negative	1.912 (−0.091 to 3.915)	0.061	2.204 (0.471 to 3.937)	0.013	0.292 (−2.308 to 2.892)	0.826
Mutation positive, asymptomatic	4.196 (2.101 to 6.292)	<0.001	1.211 (−0.656 to 3.079)	0.204	−2.985 (−5.750 to −0.220)	0.034
Mutation positive, symptomatic	11.577 (8.423, 14.730)	<0.001	9.747 (7.078 to 12.415)	<0.001	−1.830 (−5.958 to 2.298)	0.385
*APOE4* Allele absent	3.152 (1.341 to 4.965)	0.001	2.920 (1.269 to 4.580)	0.001	−0.232 (−2.718 to 2.253)	0.855
*APOE4* Allele present	7.595 (4.800 to 10.389)	<0.001	3.735 (1.626 to 5.845)	<0.001	−3.860 (−7.329 to −0.391)	0.029
High HSV‐1 antibody titres/IgM						
Mutation negative	1.598 (−0.0724 to 3.268)	0.061	2.760 (0.441 to 5.078)	0.020	1.162 (−1.705 to 4.028)	0.427
Mutation positive, asymptomatic	2.506 (0.754 to 4.257)	0.005	2.606 (0.44 to 5.078)	0.024	0.101 (−2.670 to 2.872)	0.943
Mutation positive, symptomatic	10.121 (7.625 to 12.626)	<0.001	11.275 (7.570 to 14.980)	<0.001	1.155 (−3.342 to 5.651)	0.615
*APOE4* Allele absent	2.363 (0.952 to 3.774)	0.001	4.469 (2.401 to 8.417)	<0.001	2.106 (−.310 to 4.522)	0.088
*APOE4* Allele present	6.232 (4.048 to 8.427)	<0.001	3.247 (0.493 to 6.000)	0.021	−2.986 (−6.543 to 0.572)	0.100

^1^ Mean rate of change in whole brain atrophy, mL per year.

^2^ Effect estimate is the difference in mean rates of change in whole brain atrophy (mL per year) for the exposure versus unexposed group.

#### HSV‐1 seropositivity and change in whole brain atrophy

Whole brain atrophy rates were greatest overall among mutation‐positive, symptomatic individuals. However, there was no significant difference in the mean rate of atrophy between those who were HSV‐1 seropositive and seronegative in this group (mean difference: −1.830 mL per year (95%CI: −5.958 to 2.298; *p* = 0.385). Similarly, no difference in whole brain atrophy rates was seen among mutation‐negative individuals who were HSV‐1 seropositive compared to those who were seronegative (mean difference: 0.292 mL per year (95%CI: −2.308 to 2.892; *p* = 0.826)).

Among mutation‐positive, asymptomatic participants, however, the mean rate of atrophy was greater for those who were HSV‐1 seronegative (4.196 mL per year (2.101 to 6.292)) compared to those who were seropositive (1.211 mL per year (−0.656 to 3.079)), which represented a significant difference: 2.985 mL per year (95%CI: −5.750 to −0.220; *p* = 0.0334).

Finally, among *APOE4* allele carriers, the mean rate of atrophy was greater for those who were HSV‐1 seronegative (7.595 mL per year (95%CI: 4.800 to 10.389; *p* < 0.001)) compared to those who were seropositive (3.735 mL per year (95% CI: 1.626 to 5.845, *p* < 0.001)); difference in mean atrophy rate: −3.860 mL per year, 95%CI: −7.329 to −0.391; *p* = 0.029). No such difference was seen among individuals without the *APOE4* allele between those who were seropositive and seronegative: difference in mean rate of change: −0.232 mL per year, 95%CI: −2.718 to 2.253; *p* = 0.855.

#### High HSV‐1 antibody titres/IgM and change in whole brain atrophy

There were no significant differences in the mean rate of whole brain atrophy between those who had high HSV‐1 antibody titre/IgM and those who did not, in any of the mutation and symptom status or *APOE4* allele strata (Table 5).

## Discussion

This longitudinal analysis of a sample of the DIAN cohort showed no overall association between baseline HSV‐1 seropositivity, indicating previous HSV‐1 infection, and subsequent rate of decline in the MMSE score as a global measure of cognitive function, or rate of whole brain atrophy measured by BSI. Having high HSV‐1 IgG titres or IgM at baseline, indicating recent HSV‐1 infection or reactivation, was associated with a slightly more rapid decline in the MMSE score overall, but not with rate of whole brain atrophy. In stratified analyses, while ADAD mutation carriers who were symptomatic at baseline had higher rates of decline than asymptomatic mutation carriers and non‐carriers on both MMSE score and whole brain atrophy, this was not influenced by HSV‐1 status. Among asymptomatic mutation carriers, rates of decline on both MMSE score and whole brain atrophy were slightly greater among those who were HSV‐1 seronegative. Among mutation negative individuals, no differences were seen by HSV‐1. Analyses stratified by *APOE4* status showed inconsistent results.

A number of community cohorts measuring HSV‐1 antibodies in midlife or older age have found no association with longitudinal cognitive decline or AD risk.^7^, ^8^, ^19^ While we found some limited evidence of association between high HSV‐1 titres/IgM and rate of decline on MMSE as a surrogate marker of global cognitive function, the small effect size and lack of association with rate of whole brain atrophy, is consistent with studies suggesting that any association between HSV‐1 infection and cognitive decline, if present, is likely to be subtle.^9^ We also found no evidence that either HSV‐1 seropositivity or having high titres or IgM interacted with the presence of ADAD mutations to increase rates of cognitive decline or whole brain atrophy. To our knowledge, this is the first study to investigate the interactions between past and recent HSV‐1 infection or reactivation and cognitive and brain imaging markers in this rare subgroup of ADAD families. Some previous studies of ageing cohorts have investigated the relationship between HSV‐1, *APOE4*, the most common genetic risk allele for late‐onset AD, and cognitive decline or neuroimaging markers. These have tended to show a more rapid rate of decline with HSV‐1 in *APOE4*‐positive individuals.^14^, ^15^ When we stratified by *APOE4* allele status in our specialised population, results were inconsistent: high HSV‐1 IgG titres/IgM were associated with a more rapid decline in MMSE score only among *APOE4*‐negative participants; in contrast, being seronegative for HSV‐1 was associated with a higher whole brain atrophy rate among *APOE4* carriers. Population differences might explain these inconsistent findings: the presence of the *APOE4* allele is unlikely to confer the additional level of AD risk in this very high‐risk young cohort compared to an older, more general population.

Our findings, including the lack of interaction with ADAD mutations studied, suggest that a major causal role for HSV‐1 infection on cognitive decline or whole brain atrophy in this high‐risk population is unlikely. Two recent Mendelian randomisation studies, which use genetic indicators of clinical HSV‐1 reactivation (e.g. cold sores) thereby excluding reverse causation, also found no association between genetically predicted HSV‐1 status and either cognitive function^20^ or AD risk.^20^, ^21^ While other longitudinal studies have attempted to use HSV‐1 phenotypes derived from electronic health records to investigate associations with incident dementia, there is potential for major misclassification of HSV‐1 in routine health data due to the spectrum of symptoms experienced and differences in health‐seeking behaviour.^22^ The uncertain validity of exposure definitions in these studies, including combining consultations for HSV‐1 and herpes zoster,^23^ limit their ability to test specific hypotheses about HSV‐1 and dementia. Recent robust findings replicated across four Northern European population‐based cohorts stratified by herpes subtype suggest little evidence for an association between EHR‐defined HSV‐1 infections and incident dementia risk.^24^

The DIAN cohort comprises a unique international population of families with genetic mutations causing early‐onset AD. The relatively young age of the cohort and lack of comorbidities enable questions about the aetiology and progression of AD to be addressed with fewer concerns about confounding by other clinical factors than in traditional studies of ageing. Moreover, mutation‐negative siblings provide an ideal control group. We used a quantitative HSV‐1 assay that was previously validated in a pilot study (data not shown). The availability of genotyping data enabled us to explore interactions with some genetic AD risk factors. Unlike some other studies of cross‐sectional associations between HSV‐1 and cognitive or neuroimaging outcomes, we focussed on longitudinal analysis to reduce the risk of reverse causation.

The MMSE is widely used as a brief screening tool to measure global cognitive function in both clinical practice and research studies. It has moderate to high levels of reliability, particularly test–retest reliability, making it a practical tool to track global cognitive function over time.^25^ MMSE scores correlate highly with those obtained from other cognitive screening tests^25^ as well as neuropathology, such as white matter hyperintensity volume.^26^, ^27^ While MMSE scores are affected by demographic factors such as age and education, we controlled for these in our analysis. Brain atrophy rates in the DIAN cohort are pathologically increased up to 7 years before the age of expected dementia onset, suggesting that whole brain atrophy is useful for tracking early disease progression in this high‐risk cohort.^28^

There were several limitations in our study. The DIAN cohort is a young, high‐risk cohort and, although familial AD acts as a model of late‐onset AD, findings may not generalise to other populations. The sample size was relatively small, especially when confined to individuals with at least two outcomes measured after serum samples, which limited the power for stratified analyses. While mutation‐positive asymptomatic individuals may comprise a heterogeneous group in terms of their expected time to AD onset, we lacked power to explore any differences in the effect of HSV‐1 on longitudinal outcomes among individuals with different expected times to AD onset. We used serum HSV‐1 seropositivity and high IgG titres or IgM to measure exposure. While higher IgG titres have been shown among individuals with more frequent peripheral HSV‐1 recurrences, for example cold sores,^29^, ^30^ the extent to which they reflect CNS reactivation of HSV‐1 remains unclear. Nevertheless, there is increasing evidence of greater blood–brain‐barrier permeability with age,^31^ and central HSV‐1 infection, for example in HSV encephalitis results in raised peripheral antibody titres. In this study, we did not have access to clinical data on HSV‐1 encephalitis history and did not examine CSF for HSV‐1, for example using PCR due to the very low predicted sensitivity.

We also did not have serology data on other pathogens such as CMV, which has been shown to be associated with faster rates of cognitive decline in longitudinal community studies. Two other cohorts which included HSV‐1 seropositivity as part of an infectious burden index along with HSV‐2 and CMV^11^ and HSV‐2, CMV, *Helicobacter pylori* and *Chlamydia pneumoniae* ^12^ showed that a higher infectious burden was associated with cognitive decline among older people. Despite in vitro findings showing that HSV‐1 infection stimulates a marked seeding of amyloid‐β, this association has been demonstrated with other herpesviruses as well,^1^, ^2^ reflecting the non‐specific nature of the innate immune response. It is plausible that infectious burden, rather than any specific organism, is more relevant as a risk factor for cognitive decline. Future work to explore this relationship further could combine measures of pathogen burden with use of more sensitive cognitive composite measures to capture earlier stages of cognitive decline.

In conclusion, HSV‐1 seropositivity was not associated with either rate of MMSE change or rate of whole brain atrophy measured by BSI. While having high titre HSV‐1 antibodies at baseline was associated with a slightly greater decline in MMSE overall, there was no association with rate of whole brain atrophy, suggesting that overall, HSV‐1 infection does not play a major role in decline in individuals at high risk of ADAD.

## Author contributions

CWG, JMB, DS, LS, NP, BHR and MNR were involved in conception and design of the study. DS, MRF, HM, BAG, GN, EM, RJB, PRS, JHL, JCM, DMC, NCF, BHR and MNR carried out acquisition of data. CWG, SLC, JMN and SS contributed to data analysis. CWG and SLC also carried out drafting manuscript. All authors were involved in reviewing manuscript for important intellectual content and approving the final version.

## Conflicts of Interest

SS is now employed by the Clinical Practice Research Datalink (CPRD), a division of the UK Medicines and Healthcare products Regulatory Agency (MHRA), but the views expressed in this publication are his own and do not represent the official position of either the CPRD or the MHRA. JN reports research grants from Alzheimer's Research UK, National Institute of Health Research, Alzheimer's Association, UK Dementia Research Institute, Multiple Sclerosis Society, National Multiple Sclerosis Society and Medical Research Council, membership of trial/study steering committees for Queen Mary University of London, University of Warwick and Imperial College London, and participation in Data Safety and Monitoring Boards for Guy's and St Thomas' NHS Foundation Trust and the University of Bristol. DMC reports research grants from the Alzheimer's Society, UK Medical Research Council and Alzheimer's Research UK. NCF reports research grants from Biogen, Eli Lilly, Ionis and Roche and has served on a Data Safety Monitoring Committee for Biogen. MRF reports grants from Eli Lilly and Company, Avanir, Cognition Therapeutics, Otsuka, AZTherapies, Ionis, Proclara, Merck, Cyclo Therapeutics, Prothena, Longeveron‐Biorasi, SToP‐AD, F. Hoffman – La Roche, ltd/Genentech, Biogen, Green Valley, Neurotrope Biosciences, Athira, Lexeo, Kisbee, Nervive, Pinteon, Artery Therapeutics and McClena, and participation in a Data Safety and Monitoring Board for T3D, Oligomerik. PRS reports research grants from NIH, Anonymous Foundation, Roth Charitable Foundation, NHMRC (Australia), MRFF (Australia) and NSW Health, a remunerated position as Chief Executive Officer of Neuroscience Research Australia, Company Directorships of Neuroscience Research Australia Foundation, The Health‐Science Alliance, Schizophrenia Research Institute, Australian Association of Medical Research Institutes, Australia Dementia Network (ADNeT) Ltd and StandingTall Pty Ltd and positions as President of Australasian Neuroscience Society, Steering Committee membership of Maridulu Budyari Gumal ‐ Sydney Partnership for Health Education, Research and Enterprise (SPHERE), Chair of National Medical Advisory Panel of the Judith Jane Mason & Harold Stannett Williams Memorial Foundation and Ambassador for Business Events Sydney. RJB reports research grants from the National Institute on Ageing, Avid Radiopharmaceuticals, Janssen, Hoffman La Roche/Genentech, Eli Lilly & Co, Eisai, Biogen, Abbvie and Bristol Myers Squibb, an equity ownership interest in C2N Diagnostics and royalty income based on technology (stable isotope labelling kinetics and blood plasma assay) licenced by Washington University to C2N Diagnostics, receipt of honoraria for lectures from the Korean Dementia Association and American Neurological Association, reimbursement for travel expenses for attending Alzheimer's Association roundtable and Duke Margolis Alzheimer's roundtable, participation as unpaid advisory board member for Roche Gantenerumab Steering Committee, UK Dementia Research Institute at University College London, Stanford University Next Generation Translational Proteomics for Alzheimer's and Related Dementias and Biogen – Combination Therapy for Alzheimer's Disease and receipt of income from C2N Diagnostics for serving on scientific advisory board. JCM reports grants from NIH (P30 AG066444; P01AG003991; P01AG026276; U19AG032438), consulting fees from the Barcelona Brain Research Center and TS Srinivasan Advisory Board, Chennai, India, honoraria for lectures (Montefiore Grand Rounds, NY and Tetra‐Inst ADRC seminar series, Grand Rounds, NY) and participation on a Data Safety Monitoring Board/Advisory Board for Cure Alzheimer's Fund, Research Strategy Council. HM reports funding support from AMED (17929884). EM reports grants from NIA, Hoffman‐LaRoche and Eli Lilly, payment from Eisai for continuing medical education activity, support for attending meetings from the Alzheimer Association and Alnylam, participation on a Data Safety Monitoring Board/Advisory Board for Eli Lilly, NIA and Alector, and leadership or fiduciary roles with the Fondation Alzheimer and Amzamend. MR reports NIHR grant funding to the Biomedical Research Centre and WashU DIAN funding via UCL Institute of Neurology, consulting fees for advice on Alzheimer's disease paid to UCL from Eisai and PPF axon, and an unpaid role as Chairman of DZNE senate. CWG, SLC, JMB, DS, NP, LS, BAG, GN, JHL and BR report no conflicts.

## Supporting information


**Table S1** Full models for the association between (i) HSV‐1 seropositivity, (ii) high antibody titres/IgM and the rate of change in MMSE scores.
**Table S2** Full models for the association between (i) HSV‐1 seropositivity, (ii) high antibody titres/IgM and the rate of whole brain atrophy (defined using mean BSI).
