# Supplementary material for: Herpes simplex virus and rates of cognitive decline or whole brain atrophy in the Dominantly Inherited Alzheimer Network
Source: Ann Clin Transl Neurol. 2022 Oct 3;9(11):1727–38. doi: 10.1002/acn3.51669 (PMC9639627; doi:10.1002/acn3.51669)
Supplement: Supplementary file 1 — Table S1 Full models for the association between (i) HSV‐1 seropositivity, (ii) high antibody titres/IgM and the rate of change in MMSE scores. Table S2 Full models for the association between (i) HSV‐1 seropositivity, (ii) high antibody titres/IgM and the rate of whole brain atrophy (defined using mean BSI). [file ACN3-9-1727-s001.docx]

| **Analysis** | **Variable** | **Fully adjusted effect estimate (95% C.I.*)** |
| --- | --- | --- |
| Exposure 1: HSV-1 seropositivity | HSV-1 seropositivity  - negative  - positive | Ref  0.021 (-0.438 to 0.520)** |
|  | Age category (years)  - <30 years  - 30-35  - 35-40  - 40-45  - 45-50  - >50 years | Ref  0.049 (-1.113 to 1.512)  0.107 (-0.168 to 1.026)  -0.636 (-2.490 to 0.287)  -0.404 (-1.806 to 0.715)  -1.139 (-2.928 to -0.291) |
|  | Sex  - Female  - Male | Ref  -0.672 (-1.864 to 0.246) |
|  | Education  - Below high school  - Graduated high school  - Some college education  - Bachelor’s degree or above | Ref  1.617 (-1.264 to 4.228)  1.832 (-0.713 to 3.75)  2.684 (0.174 to 4.791) |
|  | Smoking  - Never smoked  - Ever smoked | Ref  -0.259 (-1.361 to 0.621) |
|  | Race  - White  - Non-White | Ref  -1.620 (-3.224 to -0.176) |
| Exposure 2: High antibody titres/IgM | HSV-1 high antibody titres/ IgM - absent  - present | Ref  -0.365 (-0.958 to -0.072) |
|  | Age category (years)  - <30 years  - 30-35  - 35-40  - 40-45  - 45-50  - >50 years | Ref  -0.103 (-1.317 to 1.259)  -0.005 (-0.419 to 1.253)  -0.676 (-2.250 to 0.202)  -0.525 (-2.264 to 0.472)  -1.247 (-3.054 to -0.439) |
|  | Sex  - Female  - Male | Ref  -0.790 (-2.060 to 0.083) |
|  | Education  - Below high school  - Graduated high school  - Some college education  - Bachelor’s degree or above | Ref  1.681 (-1.203 to 4.922)  1.955 (-0.679 to 4.437)  2.962 (0.509 to 5.464) |
|  | Smoking  - Never smoked  - Ever smoked | Ref  -0.174 (-1.215 to 0.813) |
|  | Race  - White  - Non-White | Ref  -1.638 (-3.324 to -0.233) |

**Supplementary table 1** Full models for the association between (i) HSV-1 seropositivity, (ii) high antibody titres/ IgM and the rate of change in MMSE scores

*Bias corrected and accelerated confidence interval

**Effect estimate is the difference in mean rates of change in MMSE (points per year) for the exposure versus unexposed group adjusted for all other variables

| **Analysis** | **Variable** | **Fully adjusted effect estimate (95% C.I.*)** |
| --- | --- | --- |
| Exposure 1: HSV-1 seropositivity | HSV-1 seropositivity  - negative  - positive | Ref  -1.174 (-3.230 to 0.892) |
|  | Age category (years)  - <30 years  - 30-35  - 35-40  - 40-45  - 45-50  - >50 years | Ref  2.445 (-0.742 to 5.633)  4.449 (0.967 to 7.931)  2.817 (-0.580 to 6.213)  4.020 (0.884 to 7.155)  5.000 (1.894 to 8.107) |
|  | Sex  - Female  - Male | Ref  -0.066 (-2.169 to 2.036) |
|  | Education  - Below high school  - Graduated high school  - Some college education  - Bachelor’s degree or above | Ref  -0.204 (4.695 to 4.287)  -0.297 (-4.809 to 4.214)  -2.458 (-6.746 to 1.829) |
|  | Race  - White  - Non-White | Ref  0.188 (-2.670 to 3.047) |
| Exposure 2: High antibody titres/IgM | HSV-1 high antibody titres/IgM  - absent  - present | Ref  0.703 (-1.339 to 2.745) |
|  | Age category (years)  - <30 years  - 30-35  - 35-40  - 40-45  - 45-50  - >50 years | Ref  2.386 (-0.808 to 5.581)  4.22 (0.764 to 7.684)  2.706 (-0.697 to 6.109)  3.728 (0.635 to 6.820)  4.843 (1.747 to 7.939) |
|  | Sex  - Female  - Male | Ref  -0.188 (-2.281 to 1.905) |
|  | Education  - Below high school  - Graduated high school  - Some college education  - Bachelor’s degree or above | Ref  0.014 (-4.476 to 4.503)  0.426 (-3.989 to 4.840)  -2.028 (-6.250 to 2.195) |
|  | Race  - White  - Non-White | Ref  0.319 (-2.532 to 3.170) |

**Supplementary table 2** Full models for the association between (i) HSV-1 seropositivity, (ii) high antibody titres/IgM and the rate of whole brain atrophy (defined using mean BSI).

*Effect estimate is the difference in mean rates of change in BSI (ml per year) for the exposure versus unexposed group adjusted for all other variables. Note positive values represent greater rates of decline.
